# 

**Published:** 1917-04-28

**Authors:** 


### CONTENTS.

### SPECIAL UNITED STATES NUMBER.

| | PAGE |
|---|---|
| **Great Britain and the United States** ... ... | 59 |
| **The New Call to the Medical Profession** ... | 60 |
| **Hospital and Institutional News** ... ... ... | 61 |
| The Extension of Home Military Hospitals—The Closing of Civilian Hospitals in London—How the General Hospitals Stand—Military Hospital Efficiency and Army Requirements—A Hospital President's Curious Pessimism—A Big Memorial Scheme at Nottingham—A Rumour Denied—The After-Care of Discharged Soldiers—Suicide in Wartime—The Welsh Memorial and the Soldiers—Practical Philanthropy by Personal Service—Perils of Tuberculous Milk—Death of a Veteran Hospital Secretary—Memorial to a Hospital Treasurer—The Supervision of the Conditions in which V.A.D. Workers Live—The Overworked Probationer—A Bed Endowed by Joint Family Generosity—A Competition for Soldier Patients—A Sketch in a *Gazette*—The Cost of Afternoon Tea in Hospitals—Should Bad Meat be Sterilised?—This Week's Drug Market. | |
| **Unveiling of Bas-Relief of Florence Nightingale** ... ... ... ... ... ... ... | 66 |
| **The Service of Dedication at St. Paul's** ... | 67 |
| A Momentous Event in the World's History. | |
| **From Across the Seas** ... ... ... ... | 68 |
| **Some Great Hospitals of America** ... ... | 69 |
| British Types and New Departures. | |
| **America's Awakening to Hospital Values** ... | 71 |
| The Spread of Efficiency. | |
| **Where America Leads Great Britain** ... ... | 72 |
| **The Pay Hospital System in Great Britain** ... | 72 |
| It Should Become Widespread After the War. | |
| **The Royal College of British Nursing** ... ... | 73 |
| Its Work, Its Ambitions, and Its Outlook. | |
| **Nursing in Ireland: The Guinea-Pigs and the Mansion House Cat** ... ... ... ... ... | 74 |
| **The Matrons' and Sisters' Department** ... | 76 |
| Nursing Progress in America. | |
| Round the Hospitals. | |
| Great Speech by Hon. A. Stanley, M.P.—The College Endowment Fund Organising—The Irish Board Completed—That Lady's Umbrella—A Flagrant Misrepresentation—Nursing Affairs in Ireland. | |
| **Matrons' and Sisters' Appointments** ... ... | 77 |
| **Gifts and Bequests** ... ... ... ... ... | 77 |
| **The Editor's Letter-Box** ... ... ... ... | 78 |
| The Irish Medical Association. | |
| **The Book Market** ... ... ... ... ... | 78 |
| **Parliament and the Profession** ... ... ... | 78 |
| **Passing Events and Topics...** ... ... ... | 78 |
| **The Institutional Worker** ... ... (Suppl.) | 1 |
| Prevention of Waste in Foodstuffs: Answers to March Question. | |

